# Mental health care use in adolescents with and without mental disorders

**DOI:** 10.1007/s00787-015-0754-9

**Published:** 2015-08-15

**Authors:** Frederike Jörg, Ellen Visser, Johan Ormel, Sijmen A. Reijneveld, Catharina A. Hartman, Albertine J. Oldehinkel

**Affiliations:** University Medical Center Groningen, University Center Psychiatry, University of Groningen, House Code CC 72, PO Box 30.001, 9700 RB Groningen, The Netherlands; University Medical Center Groningen, Department of Health Sciences, University of Groningen, PO Box 30.001, 9700 RB Groningen, The Netherlands

**Keywords:** Adolescents, Health services needs and demand, Mental disorders, Cohort study

## Abstract

The aim of the study was to estimate the proportion of adolescents with and without a psychiatric diagnosis receiving specialist mental health care and investigate their problem levels as well as utilization of other types of mental health care to detect possible over- and undertreatment. Care utilization data were linked to psychiatric diagnostic data of 2230 adolescents participating in the TRAILS cohort study, who were assessed biannually starting at age 11. Psychiatric diagnoses were established at the fourth wave by the Composite International Diagnostic Interview. Self-, parent- and teacher-reported emotional and behavioral problems and self-reported mental health care use were assessed at all four waves. Of all diagnosed adolescents, 35.3 % received specialist mental health care. This rate increased to 54.5 % when three or more disorders were diagnosed. Almost a third (28.5 %) of specialist care users had no psychiatric diagnosis; teachers gave them relatively high ratings on attention and impulsivity subscales. Diagnosed adolescents without specialist mental health care also reported low rates of other care use. We found no indication of overtreatment. Half of the adolescents with three or more disorders do not receive specialist mental health care nor any other type of care, which might indicate unmet needs.

## Introduction

In recent years, the use of child and adolescent mental health care (MHC) has risen dramatically, in the Netherlands by 70 % between 2003 and 2009 [1]. Similar trends have been observed elsewhere [2–4]. Additionally, a substantial number of MHC users do not seem to have a formal DSM-IV diagnosis, especially in high-income countries [5]. It is unknown to what extent children and adolescents receive specialist MHC without having a psychiatric diagnosis. Paradoxically, only one quarter to one third of adolescents with a psychiatric diagnosis actually receives specialist MHC [6–8]. Thus, although the rise in MHC utilization among adolescents might imply overtreatment, the fact that only a limited part of the adolescents with a psychiatric disorder receives treatment suggests serious unmet needs. This study aims to investigate which of the two seems more likely.

Research into help-seeking among adolescents is mostly conducted among patients of general practitioners, where screeners establish psychological distress rather than psychiatric diagnoses [9]. In large population studies that use standardized diagnostic instruments, for instance the WHO mental health survey [5], adolescents are not included, with few exceptions [10–12]. Furthermore, aforementioned studies into MHC use relied on retrospective self-report, which can be affected by recall bias [13]. Administrative care utilization data of Psychiatric Case Registers (PCRs) are unaffected by recall bias, but have the drawback of covering only specialist MHC. Furthermore, PRCs usually do not provide reliable diagnostic data and do not reflect potential unmet needs of the general population as they only include people actually making use of services [14].

To be able to investigate MHC use among adolescents with and without a psychiatric diagnosis, we recently linked MHC utilization data from a PCR to psychiatric diagnostic data from adolescents participating in a longitudinal population-based survey (TRAILS) [15, 16]. Aside from diagnostic data, the TRAILS database also contains self-, parent-, and teacher ratings of emotional and behavioral problems and self-reported MHC use, offering insight into non-specialist MHC use and MHC use obtained in private practices.

The aims of the study were (1) to estimate the proportion of adolescents using lifetime and episode-specific specialist MHC for specific psychiatric diagnoses; (2) to estimate the proportion of adolescents without a psychiatric diagnosis using registered specialist MHC; (3) to investigate the level of emotional and behavioral problems of undiagnosed adolescents with registered specialist MHC, and, finally (4) to investigate whether participants with a psychiatric diagnosis but without registered specialist MHC report use of other care services.

## Materials and methods

### Participants

This study is part of the TRacking Adolescents’ Individual Lives Survey (TRAILS), a prospective cohort study of Dutch adolescents with the aim to explain the development of mental health from preadolescence into adulthood [15]. The present study involves data from four assessment waves of TRAILS, which ran from March 2001 to July 2002 (T1), September 2003 to December 2004 (T2), September 2005 to August 2008 (T3) and October 2008 to September 2010 (T4), respectively. Informed consent was obtained of all parents and children after the nature of the study had been fully explained. The study was approved by the Dutch Central Committee on Research Involving Human Subjects and is performed in accordance with the ethical standards laid down in the 1964 Declaration of Helsinki and its later amendments. TRAILS participants were selected from five municipalities in the North of the Netherlands, including both urban and rural areas. At T1, 2230 participants were enrolled in the study [response rate 76 %, mean age 11.1 (10–12), SD = 0.6, 51 % girls], of whom 96 % [*N* = 2149, mean age 13.6 (12–15), SD = 0.5, 51 % girls] participated at T2 and over 83 % [*N* = 1881, mean age 19.1 (17–20), SD = 0.6, 52 % girls] at T4. Non-response at enrollment was somewhat associated with low socioeconomic position, male gender and poor school performance but not with emotional and behavioral problems. Due to extensive recruitment efforts, TRAILS has so far been successful in recruiting and maintaining a diverse sample of adolescents, including a vulnerable subsample in terms of socio-economic position, psychopathology, academic achievement and substance use [15, 17].

### Psychiatric Case Register

We linked the TRAILS database to the Psychiatric Case Register North Netherlands (PCRNN), which registers MHC use since 2000. The register includes specialist treatment in child, adolescent and adult mental health and substance abuse service organizations in the North of the Netherlands, a catchment area of 1.7 million inhabitants. Primary (youth) MHC services are not included, nor are psychiatrists and psychologists in private practice and commercially based mental health services. Of all child and adolescent mental health treatment trajectories (age 0–20 years) registered in the North of the Netherlands by Statistics Netherlands (70/1000 inhabitants in 2009), 75 % is embedded in the PCRNN (53/1000 inhabitants, same age range) [18]. The PCRNN registers the number of ‘care events’, which can be an outpatient contact, a part-time treatment day or a clinical care day (24 h). At T3, the TRAILS participants and their parents were asked permission to link care use data to their records. We searched for a match in the PCRNN, but only for those participants for whom we had their own and their parents’ consent. A match was based on the first three letters of the last name, postal code and birth date. This allowed for a likelihood match (i.e., the probability that the TRAILS participant and the match found in the Register are the same person) of 95 %. We raised this number by checking whether participants might be one of a twin pair, whether they had self-reported MHC use, and whether they had moved and thus got new postal codes. Ultimately, we could verify all matches. Note that the TRAILS data manager in possession of the address information of TRAILS participants had no access to the PCRNN, while the PCRNN data manager had no access to the TRAILS database. The combined TRAILS–PCRNN dataset, in use for researchers, is completely anonymous.

### Measures

Psychiatric diagnoses were established by administering the Composite International Diagnostic Interview (CIDI) [19], a fully structured lay-administered diagnostic interview, at T4, when participants were 18–20 years old. In this study, the following DSM-IV disorders were included: mood disorders (bipolar I and II disorders, major depressive disorder and dysthymia), anxiety disorders (agoraphobia, generalized anxiety disorder, social phobia, specific phobia, panic disorder, separation anxiety disorder, obsessive–compulsive disorder and adult separation anxiety disorder), attention-deficit hyperactivity disorder, behavioral disorders (oppositional defiant disorder, conduct disorder) and substance use disorders (alcohol dependency, drug dependency). All diagnoses were made using organic exclusions and diagnostic hierarchy rules. Respondents reported on the age at onset of a disorder (i.e., the first time they suffered from the core symptoms of the disorder) and the recency (the last time of the disorder).

Emotional and behavioral problems were assessed at T1, T2 and T3 by the parent-reported Child Behavior Checklist (CBCL) and by the self-report version of this questionnaire, the Youth Self-Report (YSR) [20, 21]. At T4, the Adult Self-Report (ASR) [22] was administered. These questionnaires contain a list of behavioral and emotional problems, which parents or the participants themselves rate as 0 = not true, 1 = somewhat or sometimes true, or 2 = very or often true, in the past 6 months. A Total Problem Score scale was constructed as the sum of all problem behaviors, encompassing externalizing problems (aggressive behavior, rule-breaking behavior), internalizing problems (anxious/depressed, withdrawn/depressed, somatic complaints), and thought problems, attention problems and social problems. Teachers were asked to rate the problem behaviors of the participants at T1, T2 and T3 with the Teacher Checklist of Psychopathology, containing descriptions of problem behaviors corresponding to the eight syndrome scales of the CBCL and YSR [15, 23].

Parents reported on MHC use for emotional and behavioral problems of their children at all measurements waves (T1–T4). Parents could report service use from any of the following categories: primary care (general practitioner, social work, home care and physiotherapy), school services (school counseling, school mediation), youth social services (youth social care, regional youth care services), specialist MHC services (child and adolescent inpatient and outpatient services, psychiatrists or psychologists in private practice, psychiatric emergency care, and youth protection services), and alternative or human services (alternative therapist, traditional healer, self-help group, telephone help line and religious/spiritual leaders). TRAILS participants themselves reported on (mental) health care use at T4.

### Data analysis

First, to estimate the proportion of adolescents with a psychiatric diagnosis receiving registered specialist MHC, we calculated the percentages of adolescents with a DSM-IV diagnosis that had received specialist MHC as it was registered in the PCRNN. We first calculated lifetime registered care use for each disorder and then determined what percentage of adolescents received registered care in the period between onset and recency of the disorder (‘episode specific care’), to give an idea of the proportion of cases in which treatment was possibly sought for a comorbid disorder.

Second, to estimate the proportion of undiagnosed adolescents using registered specialist MHC, we calculated the percentage of adolescents that received lifetime registered specialist MHC without having a DSM-IV diagnosis.

Next, to furthermore explore why undiagnosed adolescents would receive specialist MHC, we investigated self-, parent- and teacher-reported problem levels of adolescents without a DSM-IV diagnosis but with registered specialist MHC. We compared their problem levels to the problem levels of three other participant groups and hypothesized that problem levels of the undiagnosed group would be lower than the diagnosed group with MHC but higher than the diagnosed group without specialist MHC. Specifically, we used ANOVA with planned contrasts to compare mean problem levels of adolescents with registered care but no DSM-IV diagnosis to (1) adolescents with neither a DSM-IV diagnosis nor registered care; (2) adolescents with a DSM-IV diagnosis without registered care and (3) adolescents with a DSM-IV diagnosis and registered care. For adolescents with registered care use, we used the point of entry into care (first contact) and investigated problem levels at the measurement wave closest to that point. For adolescents without registered care use, we used mean problem levels across the four (self-report) or three (parent- and teacher report) measurement waves.

Last, to further explore possible under-utilization, we investigated whether adolescents with a DSM-IV diagnosis but without registered specialist MHC had received other types of care and compared their use to health care use of the other groups of adolescents. We calculated percentages of adolescents reporting on the following types of MHC: general practitioner, school services, specialist MHC, psychiatrists or psychologists in private practice, youth social services, and alternative care/human services. We hypothesized that diagnosed adolescents without registered specialist MHC might receive care from psychologists or psychiatrist in private practices or help from school. We used *χ*^2^ tests to compare self-reported MHC use of adolescents with a DSM-IV diagnosis but without registered MHC to the other three groups of adolescents described above.

## Results

### MHC use of diagnosed adolescents

**Fig. 1:**
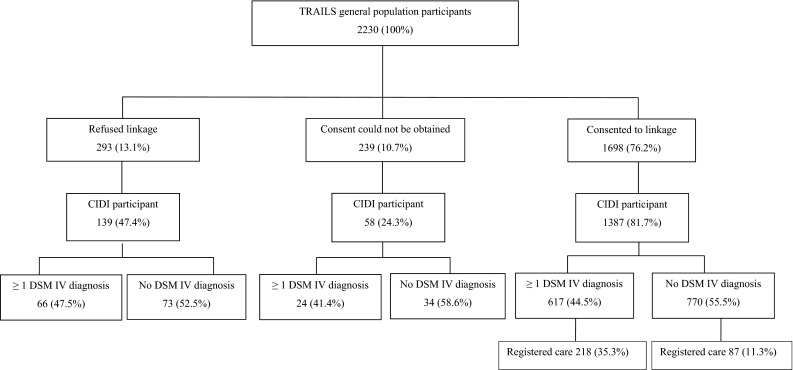
Lifetime self-reported and registered MHC use of TRAILS participants with and without one of more lifetime DSM-IV diagnoses

Approximately, one-third (35.3 %) of adolescents with any DSM-IV diagnosis received registered MHC during adolescence (Fig. 1).

Of the adolescents diagnosed with only one psychiatric disorder, 23.6 % received registered specialist MHC. This number climbed to 54.5 % when adolescents had three or more psychiatric disorders.

For each of the disorders separately, between 30 and 50 % received lifetime registered specialist MHC; only for dysthymia (60 %), bipolar disorder (62.5 %) and attention-deficit hyperactivity disorder (ADHD; 69.6 %) percentages were remarkably higher (Table 1). Furthermore, for dysthymia and ADHD approximately half of the adolescents received this care in the period between onset and recency of that disorder. For all other disorders, these percentages were much lower (Table 1).

**Table 1:** Rates of diagnosis-specific lifetime registered specialist MHC use among adolescents (*N* = 1387)

	Cases *N*	Lifetime registered care *N* (%)	Episode-specific registered care *N* (%)
No disorder	770	87 (11.3)	–
Mood disorders			
Major depressive disorder	225	105 (46.6)	41 (18.2)
Dysthymia	30	19 (63.3)	14 (46.7)
Bipolar I or II	16	10 (62.5)	4 (25.0)
Anxiety disorders			
Agoraphobia	13	7 (53.8)	5 (38.5)
Generalized anxiety disorder	58	27 (46.5)	18 (31.0)
Social phobia	177	58 (32.8)	38 (21.5)
Specific phobia	160	53 (33.1)	40 (25.0)
Panic disorder	20	9 (45.0)	5 (25.0)
Separation anxiety disorder	41	18 (43.9)	2 (4.8)
Obsessive–compulsive disorder	80	34 (42.5)	23 (28.8)
Adult separation anxiety disorder	34	16 (47.1)	7 (20.6)
Attention-deficit/hyperactivity disorder	56	38 (67.9)	28 (50.0)
Behavioral disorders			
Oppositional defiant disorder	114	55 (48.2)	30 (26.3)
Conduct disorder	106	49 (46.2)	27 (25.5)
Substance disorders			
Alcohol dependence	45	19 (42.4)	7 (15.6)
Drug dependence	59	27 (45.8)	7 (11.9)
Any disorder			
1 disorder	296	70 (23.6)	
2 disorders	165	63 (38.2)	
≤3 disorders	156	85 (54.5)	

Episode-specific registered care indicates the number (%) of adolescents that received registered specialist MHC in the period between onset of the disorder and ‘recency’ of the disorder, i.e., last period of complaints of the disorder

### MHC use of adolescents without a diagnosis

Of adolescents without a DSM-IV diagnosis, 11.3 % (*N* = 87) received registered specialist MHC (Table 1; Fig. 1). Conversely, of all MHC users, 28.5 % (87/305, see Fig. 1) had no DSM-IV diagnosis. Self-, parent- and teacher-reported problem levels for this group differed significantly from the other groups (Table 2). Contrast tests revealed that the means of parent- and teacher-reported problems were significantly higher than those of the no diagnosis and no MHC group (parent: *t* = 6.01, *p* < 0.001; teacher: *t* = 5.27, *p* < 0.001), whereas self-reported problems were slightly elevated (*t* = 2.07, *p* = 0.041) (Table 2). Compared to the group with both diagnosis and registered MHC, the group without diagnosis but with MHC had significantly lower means on self-reported problems (*t* = −5.67, *p* < 0.001), but equally high teacher-reported problem levels (*t* = −1.12, n.s.) (Table 2). Teachers rated these adolescents especially high on the subscales Attention Problems and Impulsivity.

**Table 2:** Mean behavioral and emotional problem scores for adolescents with and without specialist MHC

	No DSM-IV diagnosis No registered care *N* = 683 *M* (SD)	No DSM-IV diagnosis Registered Care *N* = 87 *M* (SD)	DSM-IV diagnosis No registered care *N* = 399 *M* (SD)	DSM-IV diagnosis Registered care *N* = 218 *M* (SD)	*F*	*p* value
Self-reported problems^a^	0.26 (0.12)	0.31 (0.20)^b^	0.37 (0.14)	0.47 (0.23)	108.40	<0.0001
Parent-reported problems	0.15 (0.10)	0.29 (0.19)^b^	0.20 (0.12)	0.35 (0.20)	110.12	<0.0001
Teacher-reported problems	0.22 (0.15)	0.51 (0.35)^b^	0.29 (0.17)	0.59 (0.38)	61.45	<0.0001

^a^‘Problems’ is defined as the total problem score derived from the Youth Self Report (self-report), Child Behavior Checklist (parent report) and the Teacher’s Checklist of Psychopathology (teacher report), measuring emotional and behavioral problems. In the current sample, total problem scores were available for 80.7 % (parent report), 87.2 % (self-report) and 33.7 % (teacher report) of the participants, respectively

^b^The overall *F* ratio is used to compare the variance between the groups to the variance within the groups. The larger the ratio, the higher is the probability that the groups differ from each other. The *F* ratio does not tell which groups are different from the others. We performed (post hoc) contrast test to compare the means of the group without diagnosis with care to the group without diagnosis without care and to the group with diagnosis with care, respectively. Results of the contrast tests are in the text

To find out more about possible reasons for treatment, we checked post hoc whether the PRCNN had registered diagnostic information of these 87 adolescents, acknowledging the reliability of these diagnoses cannot be determined. Diagnostic information in the PCRNN was present for 63 adolescents. Diagnoses included ADHD (*n* = 20), ‘parent–child problems’ (*n* = 15), anxiety (*n* = 14), adjustment disorder (*n* = 14), ‘other psycho-social circumstances’ (*n* = 10), pervasive developmental disorder (*n* = 10), oppositional defiant disorder (*n* = 7), personality disorder (*n* = 5), conduct disorder (*n* = 3), enuresis (*n* = 3) eating disorder (*n* = 3), and mental retardation (*n* = 3). Most adolescents had multiple diagnoses.

### Service use of diagnosed adolescents without specialist MHC

Of all diagnosed adolescents without registered MHC, 25.2 % reported that they did not consult any professional MHC provider during the four measurement waves (Table 3). Care from psychiatrists or psychologists in private practice, a type of specialist care not included in the PCRNN, was reported by 19.0 %. This percentage was significantly lower (*χ*^2^ = 26.5, *df* 1, *p* < 0.0001) than that of adolescents with a diagnosis who did use registered MHC, of whom 37.9 % received treatment from a professional in a private practice (in addition to PCRNN registered care). Overall, the group of adolescents with a DSM-IV diagnosis without registered MHC reported significantly less MHC use of all types (i.e., GP, help at school, specialist MHC and youth social services, all *p* values <0.0001) than the group of adolescents with a DSM-IV diagnosis with registered MHC. The proportion of self-reported alternative care use was low in both groups (*χ*^2^ = 1.4, *df* 1, n.s.). Of note is the fact that only 179 (141 + 38; Table 3) out of 305 adolescents (58.7 %) with registered specialist MHC reported to have received this type of treatment.

**Table 3:** Self-reported MHC use for adolescents with and without a psychiatric disorder and registered specialist treatment

	No DSM-IV diagnosis + No registered care *N* = 683 *N* (%)	No DSM-IV diagnosis + Registered care *N* = 87 *N* (%)	DSM-IV diagnosis + No registered care^a^ *N* = 399 *N* (%)	DSM-IV diagnosis + Registered care *N* = 218 *N* (%)	*χ* ^2^ (*df*)
No MHC	199 (29.3)	8 (9.0)	101 (25.2)***	17 (7.8)	54.1 (3)***
General practitioner	458 (67.5)	70 (78.7)	267 (69.0)***	182 (83.1)	23.1 (3)***
School services	49 (7.2)	18 (20.2)	47 (11.8)***	90 (41.1)	156.9 (3)***
Psychologists/psychiatrist in private practice	36 (5.3)	21 (23.6)	76 (19.0)***	83 (37.9)	145.4 (3)***
Specialist MHC	20 (2.9)	38 (42.7)	27 (6.8)***	141 (64.4)	532.2 (3)***
Youth social services	9 (1.3)	14 (15.7)	16 (4.0)***	50 (22.8)	144.3 (3)***
Alternative care or human services	18 (2.7)	8 (9.0)	17 (4.2)	14 (6.4)	11.6 (3)**

Information on self-reported MHC was available for 99.8 % of the sample

*** *p* value <0.001; ** *p* value <0.01

^a^Post hoc *χ* ^2^ test was performed between adolescents with diagnosis and without registered specialist MHC versus adolescents with diagnosis with registered specialist MHC

## Discussion

In this population-based study, approximately one-third of adolescents diagnosed with one or more psychiatric disorder(s) received specialist MHC as registered in the PRCNN. Adolescents diagnosed with ADHD or Dysthymia were most likely to receive this care. Of the adolescents with no diagnosis, a little over ten per cent nonetheless received specialist treatment. Put differently, of all adolescent care users, almost a third had no DSM-IV diagnosis. Parent- and teacher-reported problem levels of these adolescents were higher than those of the adolescents without a diagnosis and without care, but not as high as the problem levels of adolescents with a diagnosis and care. Finally, diagnosed adolescents without specialist MHC also reported low rates of other MHC use.

The number of adolescents receiving specialist MHC for a formally diagnosed psychiatric disorder in study is highly similar to figures reported in studies from the past 45 years (8). Apparently, MHC utilization among children and adolescents is quite stable over time and across countries and health care systems. Remarkable is the consistency in utilization rates between England, The Netherlands and Australia on the one hand, with national health care systems and relatively easy access to health care, and the United States on the other hand, where—before Obamacare—large groups of people had no insurance and hence a very difficult access to health care [7, 14, 24–26]. Apparently, two thirds of adolescents with mental health problems either do not find their way to specialist MHC, or do not wish to engage in treatment. The road from mental health problems to specialist MHC needs to cross a number of barriers, such as recognition of problems by parents, recognition and referral by a general practitioner, waiting lists, earlier experiences with MHC by parents or adolescents [9]. Although these barriers might be different across countries and time periods, it is noteworthy that, apparently, in the past 45 years no health policy, educational or stigma reducing program has been able to overcome these barriers. Stigma might still have a huge impact on MHC seeking [27].

We had a specific interest in the group of adolescents receiving MHC without having a psychiatric diagnosis. MHC without the presence of a psychiatric disorder has been reported to be quite common, with estimates up to half of the (adult) care users [28]. According to the WHO Mental Health Survey, it is present in almost all countries [5]. A recent WHO study reported that most care users have 12 months or lifetime psychiatric diagnosis, or another need indicator. The study estimated that 12–17 % of those receiving MHC did not have a recent or past diagnosis nor any other indicator of need [29]. The present study contributes in showing that it also holds for adolescents. Interestingly, percentages of treated individuals without a health care need are just as evenly distributed among countries with different cultures and health care systems as the percentage of individuals not seeking treatment. Treatment users without psychiatric disorders were shown to have other health care needs, such as serious life stressors, a previous hospitalization, suicidality or multiple subthreshold disorders [28, 29]. In our study, not adolescents themselves, but the teachers reported high problem levels for these adolescents, comparable to problem levels of care users with a diagnosis. Teachers rated them particularly high on attention and impulsivity problems, and the diagnostic information from the PCRNN also pointed in that direction. ADHD is typically diagnosed earlier during childhood. Treatment might have started early on and been continued ever since, while symptoms might have decreased to subclinical levels and not been picked up by the CIDI. For most adolescents without a DSM-IV diagnosis, the PCRNN recorded serious problems, evidencing that this group indeed seems to be highly distressed, with multiple disorders, psychosocial problems and difficult family circumstances.

A second group that had our interest consisted of adolescents with a psychiatric disorder who do not use specialist MHC. These adolescents had, on average, fewer psychiatric diagnoses and thus seemed less impaired than the group who did make use of mental health services. The type of diagnosis also differed between the two groups, with the care users having a higher prevalence of behavioral disorders and the group of non-care users of various disorders in the internalizing domain. Often, adolescents with externalizing problems are referred to care by their parents. Adolescents with internalizing problems usually do not disturb their environment as much and might not have been encouraged to seek help. Further research might elucidate why these adolescents do not seek treatment and if and how they cope with their problems.

Some limitations of this study need to be considered. First, developmental disorders are not included in the CIDI, which might explain part of the percentage of care users without a formal diagnosis. However, these are rare disorders; the PRCNN indicates that personality and developmental disorders, including mental retardation and autism, might be present in 5–18 treated adolescents without a CIDI diagnosis. Second, due to the fact that we only included participants that consented to linkage to the PCRNN, the actual registered MHC use might be higher because adolescents with current or past MHC use might be more reluctant to consent to linkage. Large discrepancies seem unlikely though, as there were no statistically significant differences in absence or presence or in number of DSM-IV diagnoses between the consenters and non-consenters (Fig. 1). Last, the PCRNN provided data on MHC use since 2000. This means specialist MHC use before 2000 is not included. Lifetime MHC use of adolescents might be somewhat higher than presented; in our study, ‘lifetime’ should be taken to mean ‘during adolescence’.

The combination of self-reported and registered MHC use provided valuable information. In a Canadian study comparing self-report to registered MHC use, 75 % of recorded care users did not report this health care use themselves [13]. In our study, underreport of health care use was less dramatic, but still only 58 % of the adolescents with registered specialist MHC reported to have received this type of treatment. Furthermore, the combination of both databases showed us that MHC use outside the register was low among the adolescents without registered MHC. Another asset of this study is the diagnostic assessment by the CIDI; research has shown that in general, there is very good concordance with clinical diagnostic assessments [30]. Last, our study is one of the few to provide estimates of MHC use among adolescents from the general population, rather than from a population with increased risk as in case of samples selected from a GP’s office or treatment facilities. Clearly, a general population representative sample is a crucial asset in light of generalization of our findings, and specifically in relation to tackling questions on overtreatment versus unmet needs.

In conclusion, we found no indication of overtreatment as only a third of diagnosed adolescents actually receive MHC, and undiagnosed MHC users were reported to have a variety of serious problems. A considerable amount of adolescents with three or more disorders, however, did not use specialist MHC, or any other type of care, which might indicate unmet needs in this group.

## References

[1] Van Dijk S, Knispel A, Nuijen J (2010). Mental health services statistics.

[2] Lee NB, Fung DS, Teo J, Chan YH, Cai YM (2003). Five-year review of adolescent mental health usage in Singapore. Ann Acad Med Singap.

[3] Matsu CR, Goebert D, Chung-Do JJ, Carlton B, Sugimoto-Matsuda J, Nishimura S (2013). Disparities in psychiatric emergency department visits among youth in Hawai’i, 2000–2010. J Pediatr.

[4] Sourander A, Santalahti P, Haavisto A, Piha J, IkAheimo K, Helenius H (2004). Have there been changes in children’s psychiatric symptoms and mental health service use? A 10-year comparison from Finland. J Am Acad Child Adolesc Psychiatry.

[5] Wang PS, Aguilar-Gaxiola S, Alonso J, Angermeyer MC, Borges G, Bromet EJ, Bruffaerts R, de Girolamo G, de Graaf R, Gureje O, Haro JM, Karam EG, Kessler RC, Kovess V, Lane MC, Lee S, Levinson D, Ono Y, Petukhova M, Posada-Villa J, Seedat S, Wells JE (2007). Use of mental health services for anxiety, mood, and substance disorders in 17 countries in the WHO world mental health surveys. Lancet.

[6] Thornicroft G (2012). No time to lose: onset and treatment delay for mental disorders. Epidemiol Psychiatr Sci.

[7] Merikangas KR, He JP, Burstein M, Swendsen J, Avenevoli S, Case B, Georgiades K, Heaton L, Swanson S, Olfson M (2011). Service utilization for lifetime mental disorders in U.S. adolescents: results of the National Comorbidity Survey-Adolescent Supplement (NCS-A). J Am Acad Child Adolesc Psychiatry.

[8] Ford T (2008). Practitioner review: how can epidemiology help us plan and deliver effective child and adolescent mental health services?. J Child Psychol Psychiatry.

[9] Zwaanswijk M, Verhaak PF, Bensing JM, van der Ende J, Verhulst FC (2003). Help seeking for emotional and behavioural problems in children and adolescents: a review of recent literature. Eur Child Adolesc Psychiatry.

[10] Kessler RC, Berglund P, Demler O, Jin R, Merikangas KR, Walters EE (2005). Lifetime prevalence and age-of-onset distributions of DSM-IV disorders in the National Comorbidity Survey Replication. Arch Gen Psychiatry.

[11] Copeland W, Shanahan L, Costello EJ, Angold A (2011). Cumulative prevalence of psychiatric disorders by young adulthood: a prospective cohort analysis from the Great Smoky Mountains Study. J Am Acad Child Adolesc Psychiatry.

[12] Newman DL, Moffitt TE, Caspi A, Magdol L, Silva PA, Stanton WR (1996). Psychiatric disorder in a birth cohort of young adults: prevalence, comorbidity, clinical significance, and new case incidence from ages 11 to 21. J Consult Clin Psychol.

[13] Drapeau A, Boyer R, Diallo FB (2011). Discrepancies between survey and administrative data on the use of mental health services in the general population: findings from a study conducted in Quebec. BMC Public Health.

[14] Bijl RV, Ravelli A (2000). Psychiatric morbidity, service use, and need for care in the general population: results of The Netherlands Mental Health Survey and Incidence Study. Am J Public Health.

[15] de Winter AF, Oldehinkel AJ, Veenstra R, Brunnekreef JA, Verhulst FC, Ormel J (2005). Evaluation of non-response bias in mental health determinants and outcomes in a large sample of pre-adolescents. Eur J Epidemiol.

[16] Ormel J, Oldehinkel AJ, Sijtsema J, van Oort F, Raven D, Veenstra R, Vollebergh WA, Verhulst FC (2012). The TRacking Adolescents’ Individual Lives Survey (TRAILS): design, current status, and selected findings. J Am Acad Child Adolesc Psychiatry.

[17] Nederhof E, Jörg F, Raven D, Veenstra R, Verhulst FC, Ormel J, Oldehinkel AJ (2012). Benefits of extensive recruitment effort persist during follow-ups and are consistent across age group and survey method. The TRAILS study. BMC Med Res Methodol.

[18] Statistics Netherlands (2015) StatLine Tables electronic databank. http://statline.cbs.nl/Statweb/. Accessed 23 June 2015

[19] World Health Organization (1990). Composite international diagnostic interview.

[20] Achenbach TM (1991). Manual for the child behavior checklist/4-18 and 1991 profile.

[21] Achenbach TM (1991). Manual of the youth self-report and 1991 profile.

[22] Achenbach TM, Rescorla LA (2003) Manual for the ASEBA adult forms & profiles. University of Vermont, Research Center for Children, Youth & Families, Burlington, VT

[23] Noordhof A, Oldehinkel AJ, Verhulst FC, Ormel J (2008). Optimal use of multi-informant data on co-occurrence of internalizing and externalizing problems: the TRAILS study. Int J Methods Psychiatr Res.

[24] Burgess PM, Pirkis JE, Slade TN, Johnston AK, Meadows GN, Gunn JM (2009). Service use for mental health problems: findings from the 2007 National Survey of Mental Health and Wellbeing. Aust N Z J Psychiatry.

[25] Ford T, Hamilton H, Meltzer H, Goodman R (2007). Child mental health is everybody’s business: the prevalence of contact with public sector services by type of disorder among british school children in a three-year period. Child Adolesc Mental Health.

[26] Whiteford H, Groves A (2009). Policy implications of the 2007 Australian National Survey of Mental Health and Wellbeing. Aust N Z J Psychiatry.

[27] Corrigan PW, Druss BG, Perlick DA (2014). The impact of mental illness stigma on seeking and participating in mental health care. Psychol Sci Public Interest.

[28] Druss BG, Wang PS, Sampson NA, Olfson M, Pincus HA, Wells KB, Kessler RC (2007). Understanding mental health treatment in persons without mental diagnoses: results from the National Comorbidity Survey Replication. Arch Gen Psychiatry.

[29] Bruffaerts R, Posada-Villa J, Al-Hamzawi AO, Gureje O, Huang Y, Hu C, Bromet EJ, Viana MC, Hinkov HR, Karam EG, Borges G, Florescu SE, Williams DR, Demyttenaere K, Kovess-Masfety V, Matschinger H, Levinson D, de Girolamo G, Ono Y, de Graaf R, Browne MO, Bunting B, Xavier M, Haro JM, Kessler RC (2015). Proportion of patients without mental disorders being treated in mental health services worldwide. Br J Psychiatry.

[30] Kessler RC, Avenevoli S, Green J, Gruber MJ, Guyer M, He Y, Jin R, Kaufman J, Sampson NA, Zaslavsky AM (2009). National comorbidity survey replication adolescent supplement (NCS-A): III. Concordance of DSM-IV/CIDI diagnoses with clinical reassessments. J Am Acad Child Adolesc Psychiatry.

